# Antimicrobial activity and safety evaluation of *Enterococcus faecium* KQ 2.6 isolated from peacock feces

**DOI:** 10.1186/s12896-015-0151-y

**Published:** 2015-05-12

**Authors:** Wei Zheng, Yu Zhang, Hui-Min Lu, Dan-Ting Li, Zhi-Liang Zhang, Zhen-Xing Tang, Lu-E Shi

**Affiliations:** College of Life and Environmental Sciences, Hangzhou Normal University, 310016 Hangzhou, Zhejiang China; College of Light Industry Science and Engineering, Nanjing Forestry University, 210037 Nanjing, Jiangsu China

**Keywords:** *E. faecium* KQ 2.6, Antimicrobial activity, Safety evaluation, Antibiotics resistance, Virulence genes

## Abstract

**Background:**

The objective of this paper was to study antimicrobial activity and safety of *Enterococcus faecium* KQ 2.6 (*E. faecium* KQ 2.6) isolated from peacock feces.

**Methods:**

Agar well diffusion method was adopted in antimicrobial activity assay. Disk diffusion test was used to determine the antibiotic resistance. The identification and virulence potential of *E. faecium* KQ 2.6 were investigated using PCR amplification.

**Results:**

The results indicated that cell free supernatant (CFS) of the strain had the good antimicrobial activity against selected gram-positive and gram-negative bacteria. The biochemical characteristics of antimicrobial substances were investigated. The results indicated that the antimicrobial substances were still active after treatment with catalase and proteinase, respectively. Moreover, the stability of antimicrobial substances did not change after heat treatment at 40, 50, 60, 70 and 80°C for 30 min, respectively. The activity of antimicrobial substances remained stable at 4 and −20°C after long time storage. The antimicrobial activity of CFS was compared with that of the buffer with similar strength and pH. The inhibitory zone of the buffer was apparently smaller than that of CFS, which meant that the acid in CFS was not the only factor that was contributed to antibacterial activity of CFS. The antibiotic resistance and virulence potential were evaluated using disk diffusion test and PCR amplification. The results showed that *E. faecium* KQ 2.6 did not harbor any tested virulence genes such as *gelE*, *esp*, *asa1*, *cylA*, *efaA* and *hyl*. It was susceptible to most of tested antibiotics except for vancomycin and polymyxin B.

**Conclusion:**

*E. faecium* KQ 2.6 may be used as bio-preservative cultures for the production of fermented foods.

## Background

Enterococci belong to lactic acid bacteria (LAB), which are widespread in foods and environment. In aspect of food fermentation, it is considered that enterococci play an important role in the development of the sensory characteristics of fermentation foods such as sausages and cheeses [1]. Certain cheese-makers have suggested that enterococci can be utilized as starter cultures in the production of Mediterranean cheese [2,3]. Furthermore, some enterococcal strains have been successfully used as preservatives to inhibit the growth of food spoilage microorganisms. One of reasons that these enterococcal strains with antimicrobial activity, produce lactic acid [4]. Lactic acid reduces the pH that can cause the disruption of cellular substrate transport systems through altering the cell membrane permeability or collapsing the electrochemical proton gradient [5]. In addition, enterococci also can produce other antimicrobial substances such as hydrogen peroxide, bacteriocin and bacteriocin like inhibitory substances (BLIS). In past few years, bacteriocin has been increasingly concerned due to its diversity and novelty. Bacteriocins are ribosomally synthesized, extracellularly released low-molecular-mass peptides or proteins [6,7]. Generally, most known bacteriocins produced by *E. faecium*, are small (<10 kDa), membrane-active and unmodified peptides. One of the most obvious traits of these bacteriocins is sensitive to proteolytic enzymes. For example, enterocin A and enterocin B from *E. faecium* MMT21 are both sensitive to trypsin, proteinase K and pronase E [8-11].

Enterococci have been used safely in foods for a long history. However, in past few years, the concerns on the safety of enterococci in food or feed industries have been raised. Many studies have reported that enterococci are associated with nosocomial infections like bacteraemia, endocarditis, urinary tract infections and diarrhea [12,13]. The main reasons that cause nosocomial infections, are the resistance of the strains to a board range of antibiotics and the presence of virulence factors in the strains [14]. The multiple antibiotic resistant strains often cause serious infections which can’t be cured well. In particular, vancomycin-resistant enterococci (VRE) have produced serious problems in public health [15]. Virulence factors have been well studied in recent years, and some virulence factors have been reported in detail. The main described factors are those are involved in adhesion, damaging tissues and evasion of immune responses (capsular polysaccharides) [16]. Additionally, it should be mentioned that enterococci may acquire antibiotic resistance and virulence factors from other enterococci, since mobile genetic elements like plasmids and transposons, can contribute to the distribution of antibiotic resistance and virulence factors between enterococcal strains [17,18]. Therefore, the safety evaluation of the enterococci should be carried out before the application.

In present study, one enterococcal strain isolated from peacock feces was identified as *E. faecium* KQ 2.6 by PCR and 16S rRNA gene sequencing. Antimicrobial activity and safety of this strain was mainly studied. The production and biochemical properties of antimicrobial substances were also investigated.

## Methods

All chemicals were purchased from Sangon (Shanghai, China). Indicator strains and antibiotic-containing disks were obtained from Binhe Microorganism Reagent Co. Ltd (Hangzhou, China). Participants in the study agreed to carry out the following studies. No human subjects including human material or human data, were contained in present study.

### Bacterial isolation and identification

Peacock feces were collected in an animal centre located in Hangzhou Normal University. Ten-fold dilutions of feces in sterile water were plated onto de Man, Rogosa and Sharpe (MRS). The plates were incubated at 37°C for 24 h. Twelve of colonies were randomly picked and used for the study of physiological and biochemical characteristics. Meanwhile, the antimicrobial activity of the strains against *Escherichia Coli* was studied using the agar spot method [19]. The strains displaying an inhibition zone were selected, and maintained as stock cultures in MRS broth supplemented with 30 % (v/v) glycerol at −20°C.

Primers, 27 F (5′-AGAGTTGATCCTGGCTCAG-3′) and 1492R (5′- GGTTACCTTGTTACGACTT-3′) based on conserved regions of 16SrRNA gene were used to direct the amplification. The program consisted of: denaturation at 94°C for 5 min, then 35 cycles of 94°C for 1 min, 55°C for 1 min and 72°C for 1 min followed by a final extension at 72°C for 5 min. Amplified PCR products were separated by 1.0 % (w/v) agarose gel electrophoresis, and then purified with the StarPrep Gel Extraction Kit (GenStar, Beijing, China) according to manufacturer’s instruction. 16S rRNA gene sequencing was carried out by Sunny Biotechnology Co., Ltd (Shanghai, China).

### Antimicrobial activity assay of *E. faecium* KQ 2.6

The antimicrobial activity of *E. faecium* KQ 2.6 against pathogenic bacteria was investigated. Pathogenic bacteria included *Bacillus subtilis*, *Bacillus cereus*, *Streptococcus pyogenes*, *Staphylococcus aureus*, *Staphylococcus epidermidis*, *E. faecalis*, *Escherichia coli*, *Pseudomonas aeruginosa*, *Klebsiella pneumoniae*, *Salmonella paratyphi*, *Candida albicans* and *Aspergillus niger*. The antimicrobial assay was performed using agar well diffusion method [20]. Firstly, *E. faecium* KQ 2.6 was grown overnight in MRS broth at 37°C. Cells in the culture were discarded by centrifugation at 10, 000 g at 4°Cfor 20 min. 60 μL of indicator bacteria (final concentration of 10^8^ CFU/mL) cultured in 20 mL soft agar containing 0.80 % (w/v) agar was poured onto a solid agar plate containing 1.5 % (w/v) agar. Afterwards, wells (8 mm in diameter) were made on agar plate, and filled with 100 μL of cell free supernatant (CFS) of *E. faecium* KQ 2.6. Plates were incubated at 37°C for 24 h after being kept for 3–4 h at 4°C. Finally, the antimicrobial activity was analyzed by observing the clear zones around the wells containing CFS. The clear zones were regarded as inhibitory zones, and recorded in mm.

### Growth kinetics and antimicrobial activity of *E. faecium* KQ 2.6

100 mL of MRS broth was inoculated with 1.0 % (v/v) of the culture of *E. faecium* KQ 2.6 and incubated at 37°C. Optical density at 600 nm (OD_600_) and pH values were monitored at 2 h intervals during 24 h. The antimicrobial activity assay was also performed every two hours. To quantify the antimicrobial activity, CFS was serially diluted 2-folds and 10 μL of each dilution was added into the wells. The titer was defined as 2^n^, which is the reciprocal of the highest dilution showing inhibition of indicator strain. Thus, the arbitrary unit (AU) of antimicrobial activity per milliliter was defined as 2^n^ × (1,000 μL/10 μL) [21].

### Effect of the biochemical factors on antimicrobial activity

*E. faecium* KQ 2.6 was cultivated in MRS broth at 37°C for 16 h. CFS was obtained by centrifugation at 10,000 g at 4°C for 20 min, and used to carry out the following studies.

Antimicrobial activity of CFS at different temperatures was investigated. CFS was treated at 40, 50, 60, 70 and 80°C for 30 min and 3 h, respectively, and at 121°C for 20 min. Storage stability of CFS at 4 and −20°C for 24, 48 h, 7 days and 15 days, was also performed.

The sensitivity of antimicrobial substances towards catalase and proteinase was studied. 1.0 mL of CFS was added to 1.0 mL of 1.0 mg/mL catalase, trypsin and pepsin, respectively. Afterwards, samples were incubated at 37°C for 30 min, and heated at 95°C for 5 min.

All treated samples were tested against *Bacillus cereus* using agar well diffusion method. Each experiment was performed at least two times. In addition, the antimicrobial activity was done using hydrogen phosphate/citric acid buffer which had a similar pH and strength to CFS of *E. faecium* KQ 2.6.

### Antibiotic resistance

Disk diffusion test was used to determine the susceptibility of *E. faecium* KQ 2.6 to antibiotics [22]. Antibiotic-containing disks were those of penicillin, vancomycin, chloramphenicol, tetracycline, erythromycin, rifampicin, ofloxacin, polymyxin B and ciprofloxacin. 20 mL of MRS broth containing 1.5 % agar was seeded with 200 μL of a culture of *E. faecium* KQ 2.6 (10^6^-10^7^ CFU/mL), and poured into a plate. Then antibiotic-containing disks were added onto the plates according to the manufacturer’s instructions. Inhibition zone diameters with/without vancomycin-containing disks were measured (mm) at 37°C after 24 and 18 h incubation, respectively. According to the recommendation of Clinical and Laboratory Standards Institute (CLSI), the strain was considered to be resistant to antibiotics if the inhibition zone was equal or smaller than 16 mm for rifampicin, 15 mm for ciprofloxacin, 14 mm for penicillin, vancomycin and tetracycline, 13 mm for erythromycin and ofloxacin, and 12 mm for chloramphenicol.

### PCR for the detection of virulence genes

PCR amplification was used to detect virulence genes *gelE* (gelatinase), *esp* (enterococcal surface protein), *asa1* (aggregation substance), *cylA* (cytolysin), *efaA* (cell-wall adhesion) and *hyl* (hyaluronidase). Primers are listed in Table 1. The following PCR conditions were used: 94°C for 5 min; followed by 35 cycles of 94°C for 1 min, 52°C (for *gelE*, *efaA*), 56°C (for *cylA*, *asa1*, *esp*) and 58°C (for *hyl*) for 30 s, 72°C for 1 min; a final extension at 72°C for 5 min. The DNA from *E faecalis* ATCC 29212 (*asa1*^+^, *cylA*^+^, *gelE*^+^, *efaA*^+^ and *hyl*^+^) was used as a positive control. The amplified products were analyzed by electrophoresis on 1.0 % (w/v) agarose gels in 1× TAE buffer.

**Table 1:** **Primer pairs used for detection of virulence genes**

**Gene**	**Primers (5**′**-3**′**)**	**Size (bp)**	**References**
*gelE*	F: TATGACAATGCTTTTTGGGAT	213	[37]
	R: AGATGCACCCGAAATAATATA		
*esp*	F: AGATTTCATCTTTGATTCTTGG	510	[37]
	R: AATTGATTCTTTAGCATCTGG		
*asa1*	F: GCACGCTATTACGAACTATGA	375	[37]
	R: AAGAAAGAACATCACCACGA		
*cylA*	F: ACTCGGGGATTGATAGGC	688	[37]
	R: GCTGCTAAAGCTGCGCTT		
*efaA*	F: GACAGACCCTCACGAATA	705	[18]
	R: AGTTCATCATGCTGTAGTA		
*hyl*	F: ACAGAAGAGCTGCAGGAAATG	276	[37]
	R: GACTGACGTCCAAGTTTCCAA		

## Results

### Isolation and identification of LAB strains with antimicrobial activity

Antimicrobial activity of twelve strains isolated from peacock feces, were studied using the agar spot method. The results indicated that only two isolates had obvious antimicrobial activity against *Escherichia coli* (data not shown). According to the studies of physiological and biochemical characteristics, one of two isolates could produce gas through glucose fermentation. It was not convenient to control the fermentation process easily. Therefore, the strain with good antimicrobial activity and gas-negative property, was chosen for this study. The sequencing of the partial 16S rRNA of the strain showed 99 % homology to that of *E. faecium* 3-2-31, so it was identified as *E. faecium* KQ 2.6.

### Spectrum of antimicrobial activity

**Figure 1:**
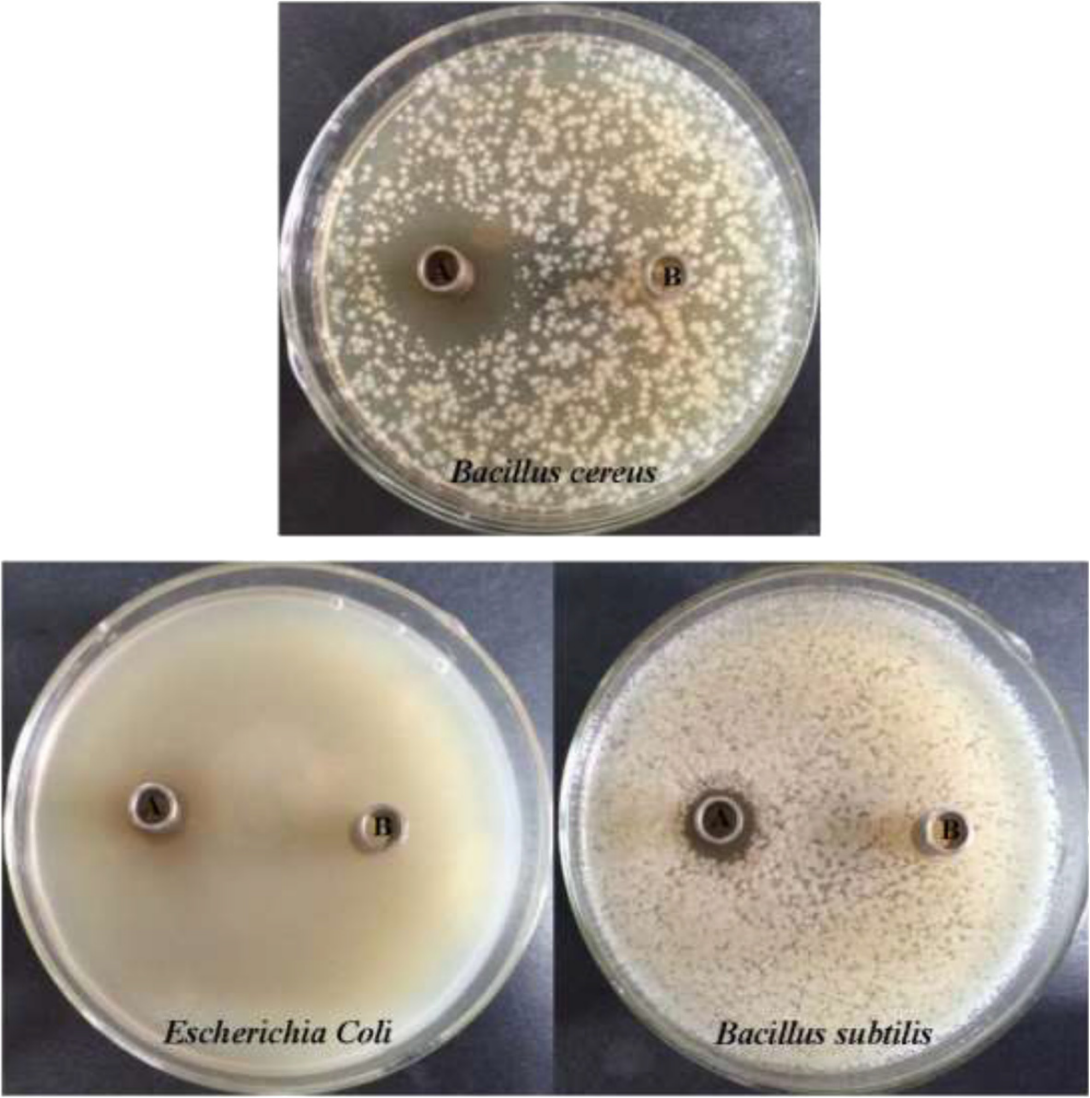
Antimicrobial activity of CFS against *Bacillus cereus*, *Escherichia coli* and *Bacillus subtilis*. A: CFS of *E. faecium* KQ 2.6, B: Luria-Bertani broth.

As shown in Figure 1, CFS of *E. faecium* KQ 2.6 could exert inhibiting activity to the growth of *Bacillus subtilis*, *Bacillus cereus* and *Escherichia coli*. The growth of a panel of pathogenic gram-positive and gram-negative bacteria including *Bacillus subtilis*, *Bacillus cereus*, *Streptococcus pyogenes*, *Staphylococcus epidermidis*, *Pseudomonas aeruginosa*, *Salmonella paratyphi* and *E. faecalis*, was also inhibited by CFS of *E. faecium* KQ 2.6. However, it was not active against fungi like *Candida albicans* and *Aspergillsu niger* (Table 2).

**Table 2:** **Antimicrobial activity of CFS produced by** ***E. faecium*** **KQ 2.6**

**Indicator strains**	**Medium**	**Incubation temperature(°C)**	**Antimicrobial activity** ^**a**^
Gram-positive			
*Bacillus subtilis*	LB	37	+
*Bacillus cereus*	LB	37	+++
*Streptococcus pyogenes*	LB	37	++
*Staphylococcus aureus*	LB	37	-
*Staphylococcus epidermidis*	LB	37	++
*E. faecalis*	MRS	37	+
Gram-negative			
*Escherichia Coli*	LB	37	+
*Pseudomonas aeruginosa*	LB	37	++
*Klebsiella pneumoniae*	LB	37	-
*Salmonella paratyphi*	LB	37	+++
Fungi			
*Candida albicans*	PDA	28	-
*Aspergillus niger*	PDA	28	-

^a^Results of antimicrobial activity were recorded in the diameter of inhibition zones around the wells (8 mm in diameter): −, no inhibition zone; +, zone < 5 mm; ++, zone < 5–10 mm; +++, zone > 15 mm.

### Production of antimicrobial substances and growth kinetics

**Figure 2:**
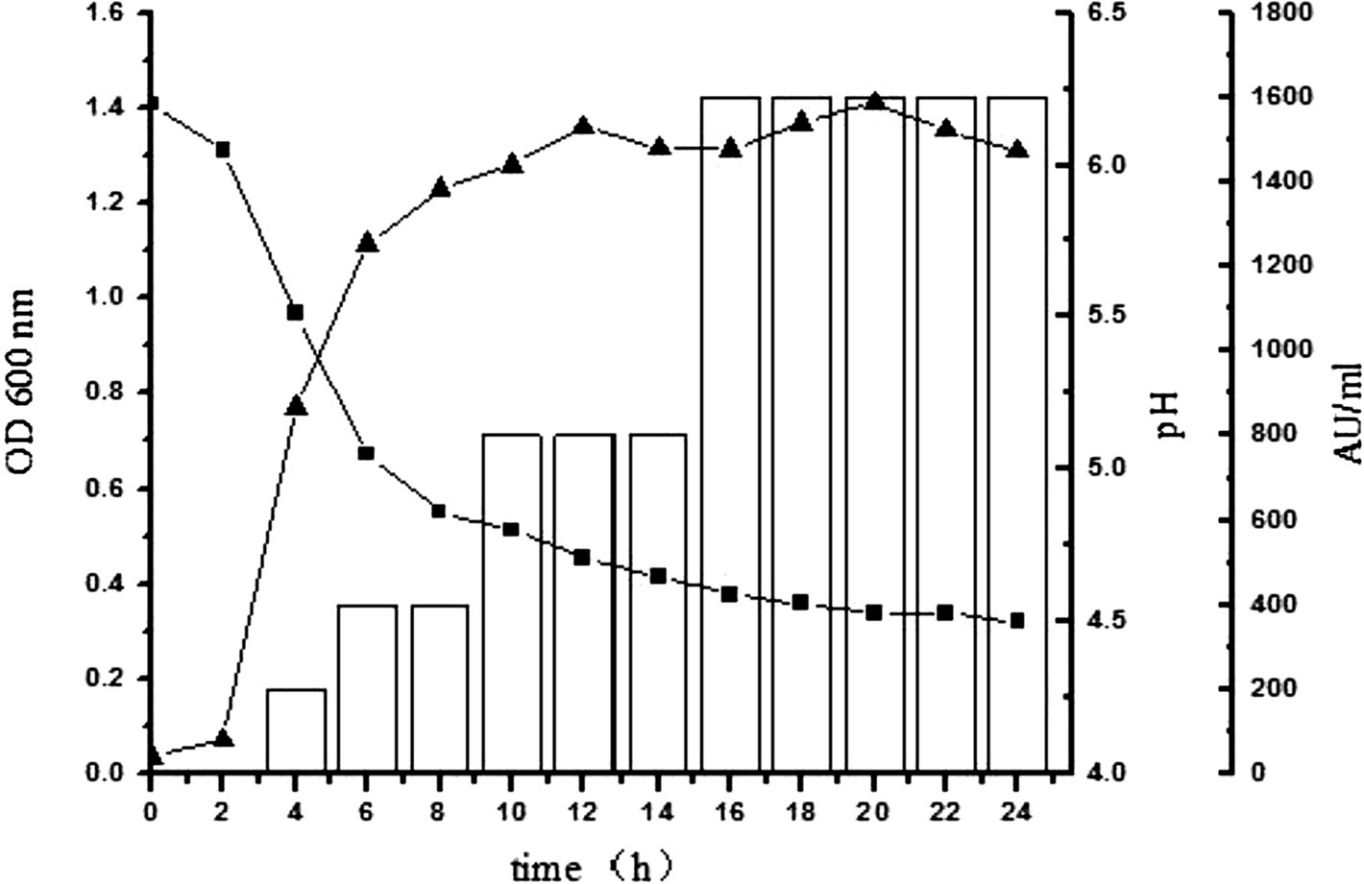
Kinetics growth curves and production of antimicrobial substances by *E. faecium* KQ 2.6. ▲: OD_600_; ■: pH of the culture medium; black histograms: antimicrobial activity against *Bacillus cereus.*

The results of the cell density, pH of the media and production of antimicrobial substances were obtained during 24 h of growth at 37°C (Figure 2). During this period, the cell density of *E. faecium* KQ 2.6 increased from 0.03 to 1.37 (OD_600_). pH of the media dropped down to 4.5. *E. faecium* KQ 2.6 began to produce antimicrobial substances (200 AU/mL) after 4 h of growth. Maximum values (1600 AU/mL) of antimicrobial activity was reached at the early stationary phase (16 h), and remained un-change in the following 8 h of growth.

### Characterization of antimicrobial substances

**Figure 3:**
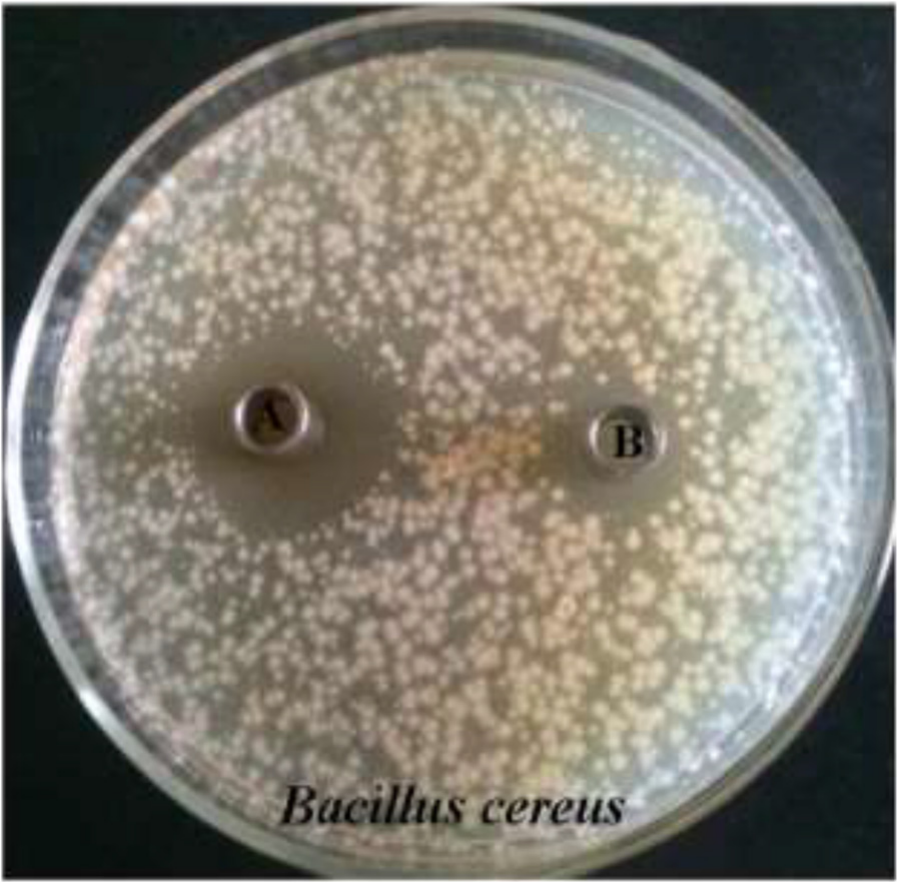
Antimicrobial activity of CFS and buffer against *Bacillus cereus*. A: CFS of *E.faecium* KQ 2.6, B: hydrogen phosphate/citric acid buffer.

Except for heat treatment at 121°C for 20 min, the substances remained stable after heating at 40, 50, 60, 70 and 80°C for 30 min, respectively. Meanwhile, antimicrobial activity did not change when CFS was stored at low temperatures(4 and −20°C) for 24, 48 h, 7 and 15 days (Table 3). It showed that storage conditions did not led to the decrease of antimicrobial activity significantly. Additionally, the addition of catalase, trypsin and pepsin to CFS had no effect on antimicrobial activity of CFS (Table 3). The inhibitory zone of hydrogen phosphate/citric acid buffer was apparently smaller than that of CFS (Figure 3).

**Table 3:** **Effect of temperature and enzymes on the activity of CFS of** ***E. faecium*** **KQ 2.6**

**Treatments**	**Antimicrobial activity** ^**a**^
Temperature	
40°C for 30 min	+
50°C for 30 min	+
60°C for 30 min	+
70°C for 30 min	+
80°C for 30 min	+
121°C for 20 min	-
Enzymes	
Catalase	+
Trypsin	+
Pepsin	+

^a^+, presence of antimicrobial activity; −, absence of antimicrobial activity; the indicator strain, *Bacillus cereus*

### Detection of antibiotic resistance and potential virulence factors

Phenotypic results from disk diffusion test demonstrated that *E. faecium* KQ 2.6 was highly susceptible to most of tested antibiotics such as penicillin, chloramphenicol, tetracycline, erythromycin, rifampicin, ofloxacin and ciprofloxacin. However, it was also found that the strain was resistant to vancomycin and polymyxin B (Table 4).

**Table 4:** **Antibiotic resistant profile of** ***E. faecium*** **KQ 2.6**

**Antibiotics**	**Drug concentration per disk (μg)**	**Susceptibility** ^**a**^
Penicillin	10	S
Vancomycin	30	R
Chloramphenicol	30	S
Tetracycline	30	S
Ofloxacin	5	S
Erythromycin	15	S
Rifampicin	5	S
Polymyxin B	30	R
Ciprofloxacin	5	S

^a^The antibiotic resistance was determined by disk diffusion test. The sensitive was analyzed by the recommendation of CLSI (2008). S: sensitive; R: resistant.

**Figure 4:**
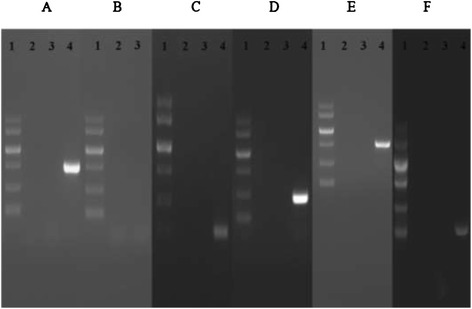
Results of *E. faecium* KQ 2.6 using primers directed against **(A)** 688 bp fragment of the *cylA* gene, **(B)** 510 bp fragment of the *esp* gene, **(C)** 213 bp fragment of the *gelE* gene, **(D)** 375 bp fragment of the *asa1* gene, **(E)** 705 bp fragment of the *efaA* gene and **(F)** 276 bp fragment of the *hyl* gene. Lane 1: standard molecular weight (2000 kb); lane 2: negative control; lane 3: *E. faecium* KQ 2.6; lane 4: positive control (*E. faecalis* ATCC 29212).

Whether the presence of virulence genes encoding *gelE*, *esp*, *asa1*, *cylA*, *efaA* and *hyl* in the strain was investigated. The results from agarose gel electrophoresis showed that *E. faecium* KQ 2.6 did not harbor virulence genes including *gelE* (213 bp), *esp* (511 bp), *asa1* (328 bp), *cylA* (688 bp), *efaA* (704 bp) and *hyl* (276 bp) (Figure 4).

## Discussion

Enterococci occur in many different environments such as in air, soil, water and the gastrointestinal tract of animals and humans. Due to the association of enterococci with the gastrointestinal tract, it is an ordinary and efficient method to screen enterococci from animal feces. In the last decades, the benefic role of enterococci from animal and human feces in food and animal industries has been well studied [1,23,24]. In this study, twelve isolates were screened from peacock feces, and two of them displayed good antimicrobial properties. The highest antimicrobial activity and gas-negative strain was named as *E. faecium* KQ 2.6.

Antimicrobial activity of *E. faecium* KQ 2.6 was evaluated. The results showed that this strain was able to inhibit gram-positive and gram-negative bacteria. It should be pointed out that many enterococci can produce bacteriocins, which exhibit activity towards gram-positive and gram-negative bacteria [25]. Therefore, the hypothesis that antimicrobial activity of *E. faecium* KQ 2.6 is due to the produced bacteriocin, may be established. However, the activity did not lost after CFS of *E. faecium* KQ 2.6 was treated by proteinase. It demonstrated that the antimicrobial factors were not protein components such as bacteriocin or BLIS. The resistance of CFS to catalase indicated that antimicrobial substance was not hydrogen peroxide. Regarding this phenomenon, some reports have been indicated that the antimicrobial activity may be due to the produced acid [26,27]. Anyogu et al. [28] also indicated that the acid substances produced by *E. faecium* was an important factor to deter the growth and survival of pathogens in the process of submerged cassava fermentation. Therefore, the antimicrobial activity of enterococci in this study may be due to the production of organic acids. Our results showed that the produced acid was not the only factor that contributed to antimicrobial activity of CFS of *E. faecium* KQ 2.6, since the inhibitory zone of CFS was significantly bigger than that of the buffer with similar pH and strength. Thus, we believed that another type of antimicrobial substance should be in CFS of *E. faecium* KQ 2.6.

To study antimicrobial substances of *E. faecium* KQ 2.6 more specially, the heat stability and storability were investigated. The activity could be kept stably after a long time storage or high temperature treatment. It indicated that storage conditions did not lead to the decrease of antimicrobial activity significantly. The high stability of antimicrobial activity can be a good criterion for its use as a bio-preservative under complicated conditions of food processing.

The incidence of antibiotic resistance has been received high attention as it is of vital point for the safe use of the strains in foods. It is clear that in hospital environment, multiple antibiotic resistant strains may lead to infections or super-infections. Enterococci are the fourth prevalent strains causing blood infections in European hospital, and the proportion of enterococcal infections continues to increase, mainly because of an increasing number of antibiotic resistant *E. faecium* [29]. In our study, *E. faecium* KQ 2.6 had resistance to vancomycin and polymyxin B. The results indirectly agreed with the study of Messi et al. [30]. Vancomycin-resistance enterococci (VRE) are not restricted to clinical strains, but can be obtained from animal organs and environment. In last few years, the numbers of VRE have been increasing [31]. VRE have brought treatment difficulty, as vancomycin is the last few therapeutic options for enterococcal infections [32,33]. The mechanism of the high resistance to vancomycin is the replacement of the terminal D-Ala of peptidoglycan precursors with D-lactate, which can prevent or destroy the combination between vancomycin and peptidoglycan precursors [34]. Fortunately, *E. faecium* KQ 2.6 was sensitive to the most common antibiotics such as penicillin, tetracycline, chloramphenicol and ciprofloxacin. Therefore, the strain was not multiple antibiotic resistant enterococci.

The investigation of antibiotic resistance alone can’t evaluate the safety of enterococci completely. Virulence factors are greatly contributed to enhance infection risks, so potential virulence genes of *E. faecium* KQ 2.6 need to be evaluated. It was reported that the genes encoding adhesion-associated protein were rarely detected in *E. faecium* strain from foods [18]. The absence of full *Cyl* operon in *E. faecium* has also been reported [31]. Our results indicated that this strain did not harbor tested virulence genes *gelE*, *esp*, *asa1*, *cylA*, *efaA* and *hyl*, which was in agreement with the above conclusions. In general, the clinical enterococci harbor more virulence factors than *E. faecium* KQ 2.6.

However, it should be noted that mobile genetic elements like plasmids and transposons, may contribute to the distribution of virulence factors between enterococcci isolated from different sources [17,18]. The virulence genes acquisitions in *E. faecium* have been reported. Clonal complex 17 lineage, a kind of *E. faecium* genetic lineage, can obtain an *esp* gene from other clinical enterococci. And this lineage not only occurs in hospital but also is found in foods [35,36]. Another study indicated that less than 40 % of *E. faecalis* proteins have been found in *E. faecium* draft genome. So, *E. faecium* may harbor additional virulence factors from *E. faecalis* [16]. Furthermore, Sex pheromones or gene transfer pheromones may promote acquisition of virulence genes from other enterococci. Even it is not a common trait that enterococci produce sex pheromones or gene transfer pheromones [18], the work on detecting the presence of sex pheromones or gene transfer pheromones will contribute to assess the safety of the strain.

## Conclusion

To our knowledge, this is the first report on the study of *E. faecium* isolated from peacock feces. *E. faecium* KQ 2.6 not only inhibited the growth of gram-positive bacteria, but also had antimicrobial activity towards gram-negative bacteria. The antimicrobial substance was not hydrogen peroxide or protein components. Part inhibitory effect of *E. faecium* KQ 2.6 might be due to the produced acid. Another antimicrobial substance should be in CFS of *E. faecium* KQ 2.6. *E. faecium* KQ 2.6 may be considered safely for its susceptibility to most common antibiotics and absence of the most studied virulence genes. Therefore, this strain has potential to be used as a food preservative in our daily life. However, it should be further evaluated for its ability of virulence genes acquisitions before this strain is applied in the food and/or feed industries.

## Abbreviation

*E. faecium* KQ 2.6: *Enterococcus faecium* KQ 2.6
*E. faecalis*: *Enterococci faecalis*
CFS: Cell free supernatant
BLIS: Bacteriocin like inhibitory substances
LAB: Lactic acid bacteria
VRE: Vancomycin-resistant enterococci
LB: Luria-Bertani broth
MRS: de Man Rogosa Sharpe agar
PDA: Potato Dextrose Agar
CLSI: Clinical and Laboratory Standards Institute
